# Identification of Replication Competent Murine Gammaretroviruses in Commonly Used Prostate Cancer Cell Lines

**DOI:** 10.1371/journal.pone.0020874

**Published:** 2011-06-17

**Authors:** Karen Sandell Sfanos, Amanda L. Aloia, Jessica L. Hicks, David M. Esopi, Jared P. Steranka, Wei Shao, Silvia Sanchez-Martinez, Srinivasan Yegnasubramanian, Kathleen H. Burns, Alan Rein, Angelo M. De Marzo

**Affiliations:** 1 Department of Pathology, Johns Hopkins University School of Medicine, Baltimore, Maryland, United States of America; 2 HIV Drug Resistance Program, National Cancer Institute, Frederick, Maryland, United States of America; 3 Department of Oncology, The Sidney Kimmel Comprehensive Cancer Center, Baltimore, Maryland, United States of America; 4 Institute of Genetic Medicine, Johns Hopkins University School of Medicine, Baltimore, Maryland, United States of America; 5 Advanced Biomedical Computing Center, Science Applications International Corporation, Frederick, Maryland, United States of America; 6 Department of Urology, The Brady Urological Research Institute, Johns Hopkins University School of Medicine, Baltimore, Maryland, United States of America; Institut Pasteur, France

## Abstract

A newly discovered gammaretrovirus, termed XMRV, was recently reported to be present in the prostate cancer cell line CWR22Rv1. Using a combination of both immunohistochemistry with broadly-reactive murine leukemia virus (MLV) anti-sera and PCR, we determined if additional prostate cancer or other cell lines contain XMRV or MLV-related viruses. Our study included a total of 72 cell lines, which included 58 of the 60 human cancer cell lines used in anticancer drug screens and maintained at the NCI-Frederick (NCI-60). We have identified gammaretroviruses in two additional prostate cancer cell lines: LAPC4 and VCaP, and show that these viruses are replication competent. Viral genome sequencing identified the virus in LAPC4 and VCaP as nearly identical to another known xenotropic MLV, *Bxv*-1. We also identified a gammaretrovirus in the non-small-cell lung carcinoma cell line EKVX. Prostate cancer cell lines appear to have a propensity for infection with murine gammaretroviruses, and we propose that this may be in part due to cell line establishment by xenograft passage in immunocompromised mice. It is unclear if infection with these viruses is necessary for cell line establishment, or what confounding role they may play in experiments performed with these commonly used lines. Importantly, our results suggest a need for regular screening of cancer cell lines for retroviral “contamination”, much like routine mycoplasma testing.

## Introduction

Xenotropic Murine leukemia virus-Related Virus (XMRV) was first reported in 2006 as a novel gammaretrovirus found in prostate tissues from prostate cancer patients [1]. Subsequent studies failed to reach a consensus on the prevalence of the virus in prostate cancer, with reported numbers ranging from 0% to approximately 27% (reviewed in [2], [3]). In 2009, it was reported that the commonly used prostate cancer cell line CWR22Rv1 contains replication competent XMRV, and produces the virus at high levels in culture [4].

It is not currently known precisely how the CWR22Rv1 cell line became infected with XMRV. One explanation is that the virus was in the prostate tumor from which the cell line was established. If proven, this discovery would support the argument that XMRV is present in humans, and can establish an infection in the human prostate. Another explanation could be that the cell line was infected after the cells were removed from the patient, for example, during xenotransplantation into immunocompromised mice (a commonly used and often necessary practice in the establishment of prostate cancer cell lines). Indeed, recent data presented by Pathak *et. al.* at the 17^th^ Conference on Retroviruses and Opportunistic Infections (CROI) provide a compelling argument that XMRV likely originated from recombination between two separate and defective endogenous MLVs that infected the CWR22 human cancer cells at some point during the process of serial passage of the cells by xenograft (see [5] for review). This xenograft was later used to produce a cell line, CWR22Rv1, which is cultivated in many laboratories throughout the world that study prostate cancer. Reports of infection of human cell lines with animal retroviruses are quite common in the literature [6], [7], [8], (reviewed in [9]) and it is well documented to occur following xenotransplantation of cells and tissues in immunocompromised mice (reviewed in [10]). There are also reports of retroviral infection of cell lines that clearly occurred during passage in culture, as the lines were never passaged in mice [9], [11]. Additional potential sources of cell line infection with retroviruses include laboratory contamination and the use of retroviral vectors in research [9]. Importantly, there are only rare examples where retroviral presence in cultured human cells could be traced back to a true infection of the patient. One well established example of this is the discovery of the human T-lymphotropic virus type 1 (HTLV-1) in cultured cells from patients with T-cell lymphomas [12].

The discovery of XMRV in the CWR22Rv1 cell line prompted us to interrogate additional prostate cancer cell lines for the presence of XMRV or other murine gammaretroviruses. If the source of retroviral infection is not from the prostate cancer patient, but introduced by passage through animals or laboratory contamination, then the presence of replication competent retroviruses in commonly used prostate cancer cell lines for research could potentially have confounding effects on experimental outcomes.

## Materials and Methods

### Cell Lines

Source of cell lines and description of cell line tissue microarray (TMA) construction is provided in the Text S1. All of the cell lines used were authenticated via short tandem repeat (STR) profiling of 9 genomic loci with the Powerplex 1.2 system (Promega) before inclusion in the TMA.

### XMRV/MLV Immunohistochemistry (IHC)

Slides containing sections of formalin-fixed paraffin-embedded (FFPE) control cells or TMAs were deparaffinized and steamed for 25 min. in citrate-based unmasking solution for antigen retrieval (Vector Laboratories). IHC (including positive and negative controls) with either anti-p30 (MLV30) or anti-gp70 (MLV70) antisera was performed as previously described [3].

### XMRV and MLV PCR

Genomic DNA (gDNA) was extracted from cultured cell pellets from each of the cell lines used in the TMA using the DNeasy Blood and Tissue Kit (Qiagen). All DNA was first amplified with genomic GAPDH PCR primers (GAPgen-F 5′-GGGCTCTCCAGAACATCATCC-3′ and GAPgen-R 5′-GTCCACCACTGACACGTTGG-3′) to verify the quality of the DNA template. MLV-specific primer (In-For) as well as the XMRV-specific reverse primer (Deletion-Rev) were as previously described [13] with an expected product size of 104 bp. The degenerate Pan-MLV primer sequences were as follows: Pan-MLV-F 5′-GCARCCCWGGGAGACGTC-3′ and Pan-MLV-R 5′-AGACRCGCRGCGCGGYT-3′ with an expected product size of approximately 206 bp (181 bp for XMRV). PCR was carried out in a total volume of 25 µl (containing 1X PCR buffer, 1.5 mM MgCl_2,_ 200 µM dNTPs, 400 nM F and R primer, 1U AmpliTaq Gold, Applied Biosystems) using approximately 100 ng of gDNA for template and the following cycling parameters: 95°C for 2 min.; 40 cycles of 95°C for 30 sec., 54°C for 1 min. (Pan-MLV primers) or 64°C for 30 sec. (XMRV-specific primers), 72°C for 30 sec.; final extension at 72°C for 7 min. Serial dilution tests of CWR22Rv1 gDNA indicated that the degenerate Pan-MLV PCR assay had a sensitivity of detecting virus in down to 0.1 ng of CWR22Rv1 gDNA (equivalent to ∼100–200 viral genomes or ∼10–20 infected cells [3], [4]) and the XMRV-specific PCR assay was capable of detecting down to 1–2 viral genomes (Figure S1). The difference in sensitivity can likely be explained in part by the use of degenerate primers for Pan-MLV PCR.

### Long-range PCR and Sequencing of Viral Genomes

Full-length or near full-length viral genomes were amplified from genomic DNA preparations of the LAPC4, VCaP and EKVX cell lines and sequenced as described in the Text S1 and Table S2.

### Phylogenetic Analysis

Full-length genomes from known endogenous MLV sequences [14] and the virus genomes isolated from LAPC4, VCaP and EKVX were aligned using ClustalW. Based on the alignment, a neighbor-joining tree was generated using the default settings of MEGA version 5 (MEGA 5) [15].

### Infectivity Assay

Cell line supernatants were collected after 24 hr. culture and filtered through a 0.45 µm filter. The infectivity assay uses iGLuc-DERSE cells, a 293 mCAT based cell line that produces the *Gaussia* Luciferase (GLuc) enzyme only after infection with a replication-competent MLV. iGLuc-DERSE cells were seeded at 3.5×10∧4 cells/well in a 12-well plate. Cells were treated with 20 µg/ml DEAE-dextran for 30 min. DEAE-dextran was then removed and cells were rinsed with media. Next, 500 µl of each supernatant was added to the iGLuc-DERSE cells in triplicate. Cells were incubated for 3 hr. and 400 µl of additional media was added. Two and three days after infection, 10 µl of the media from each well was assayed for luciferase activity. At 3 days after infection, cells were passaged. Two days after cell passage, luciferase activity was measured again. Cells were passaged for a total of 13 days. Further details of the cell line will be given in a future publication.

### Vectorette PCR

Vectorette PCR was performed as previously described [16]. Briefly, 2 µg of genomic DNA was digested overnight at the appropriate temperature with the following restriction enzymes: *Ase*I, *BspH*I, *BstY*I, *Hind*III, *Nco*I, and *Pst*I. Vectorette oligos were then annealed overnight at 4°C at a concentration of 1 µM using T4 DNA ligase (New England Biolabs). Vectorette PCR was conducted using a virus-specific forward primer (either Virus-vec-F1 5′-GACTGAGTCGCCCGGGTACC-3′, or Virus-vec-F2 5′-CGCTTCTCGCTTCTGTAACCGCG-3′, both in the 3′ LTR in nucleotide position 8525 and 8437 of Xmv43, respectively) and a vectorette-specific reverse primer (5′-CTCTCCCTTCTCGAATCGTAA-3′). PCR products were gel purified and cloned into the pCR®2.1-TOPO vector (Invitrogen). Transformed bacterial colonies were chosen at random for sequencing.

## Results

### LAPC4, VCaP and EKVX cancer cell lines are infected with a murine leukemia virus which is not XMRV

**Figure 1:**
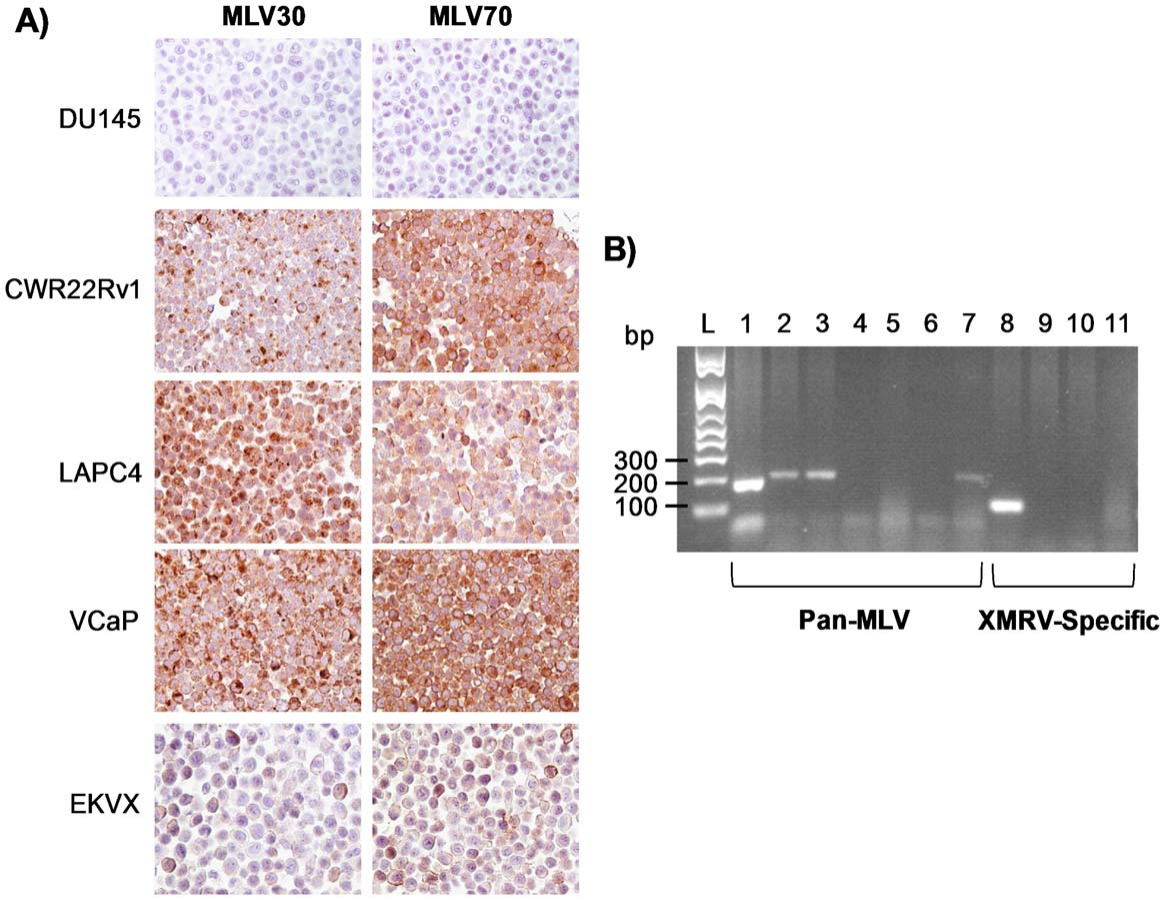
LAPC4, VCaP and EKVX cancer cell lines are infected with a murine leukemia virus which is not XMRV. (**A**) Examples of MLV-negative (DU145) and positive (CWR22Rv1, LAPC4, VCaP and EKVX) cancer cell lines stained with MLV30 and MLV70 antisera. (**B**) Positive staining in LAPC4, VCaP and EKVX does not represent XMRV. Lanes 1–7, PCR with MLV-specific primers (1 =  CWR22Rv1, 2 =  LAPC4, 3 =  VCaP, 4 =  DU145, 5 =  LNCaP, 6 =  PC3, 7 =  EKVX), Lanes 8–11, PCR with XMRV-specific primers (8 =  CWR22Rv1, 9 =  LAPC4, 10 =  VCaP, 11 =  EKVX). Difference in product size between CWR22Rv1 (lane 1) and LAPC4, VCaP or EKVX (lanes 2,3,7) is due to the XMRV-specific 24-nt deletion [13]. L =  molecular weight ladder.

As an initial screen for murine gammaretroviruses in human cell lines, we utilized a tissue microarray (TMA) containing a total of 72 cell lines, which included 58 of the 60 human cancer cell lines used in anticancer drug screens and maintained at the NCI-Frederick (NCI-60) (Table S1). As shown in Figure 1A, IHC with broadly reactive antisera for Moloney murine leukemia virus (Mo-MLV) p30^CA^ (MLV30) or gp70^SU^ (MLV70) [3] identified three prostate cancer cell lines (CWR22Rv1, LAPC4 [17], VCaP [18]) as positive for virus. While CWR22Rv1 was previously reported to be infected with replication competent XMRV [4], LAPC4 and VCaP are not known to contain retroviruses. Interestingly, in a previous study, cell culture media from VCaP was anecdotally reported to display viral activity [4]. We also identified a non-small-cell lung carcinoma cell line, EKVX, as being positive for virus.

We next used a PCR-based assay to determine whether LAPC4, VCaP and EKVX are infected with XMRV. As shown in Figure 1B, PCR with degenerate pan-MLV primers (designed to broadly amplify MLV species) identified a positive product as expected for CWR22Rv1, LAPC4, VCaP and EKVX. Also as expected based on IHC results, PCR with the degenerate MLV primers was negative for the prostate cancer cell lines DU145, LNCaP and PC3. PCR with primers that span a 24 nucleotide deletion contained in the genome of XMRV, but not in any other known MLVs [13] was only positive for CWR22Rv1 (Figure 1B), indicating that the virus(es) present in the remaining cell lines that were positive by IHC are not XMRV. The complete results of IHC and PCR on the 72 cell lines are shown in Table S1. To control for the possibility that positive PCR results may have been due to mouse DNA contamination, we tested all positive cell lines with a PCR assay for mouse intracisternal A particle (IAP) as previously described [19]. As shown in Figure S2, none of the cell lines were found to be positive for contaminating mouse DNA. The IAP assay has previously been shown to be highly sensitive for mouse DNA, and to out-perform PCR-based assays for mouse mitochondrial DNA (mtDNA) [19].

### Viral genome sequencing, phylogenetic analysis and integration site mapping

**Figure 2:**
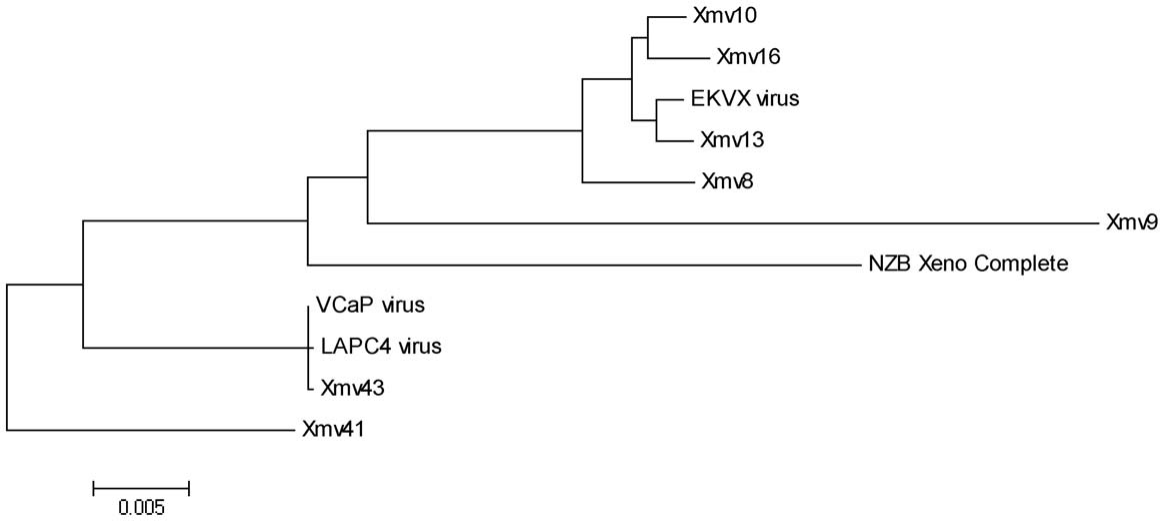
Phylogenetic analysis of virus genomes from LAPC4, VCaP and EKVX cell lines. The phylogenetic tree represents known xenotropic MLV sequences. This tree represents only xenotropic MLVs for which the full proviral sequence is known, which is likely only a small portion of all endogenous xenotropic MLVs present in mice.

In order to determine the identity of the viruses present in LAPC4, VCaP and EKVX, we performed long-range PCR and sequencing as described in the Text S1. An 8,046 nucleotide sequence (GenBank Accession #JF908817) obtained from EKVX clustered with a xenotropic MLV group in phylogenetic analysis, but was not identical to any MLVs for which the full-length genomic sequence is known (Figure 2, Supporting Figure S3). This sequence was also determined in NCBI BLAST database searches to be 98% similar to a retrovirus previously isolated from the DG-75 B-lymphoblastoid cell line [20], and identical to partial *pol* and *env* sequences isolated from EKVX in another recent study [8].

For LAPC4 and VCaP, vectorette PCR was performed to determine viral integration sites and the 5′ and 3′ ends of the viral genomes. Nearly identical (99% similar) viral genomes 8,657 nucleotides in length (GenBank Accession #JF908815, JF908816) were sequenced from the LAPC4 and VCaP cell lines. NCBI BLAST and Ensembl database searches revealed an almost perfect match (99%) to a sequence contained on Chromosome 1 of the C57BL/6J mouse genome sequence (nucleotide position 172871652–172880308). This location maps to a known murine xenotropic gammaretroviral provirus termed *Bxv*-1 (or XMV43) [21]. Phylogenetic analysis also revealed that the LAPC4 and VCaP viruses are phylogenetically identical to *Bxv*-1 (Figure 2, Supporting Figure S3). *Bxv*-1 is an intact and infectious provirus and, although commonly used prostate cancer cell lines are not known to be contaminated with this virus, *Bxv*-1 is known to be capable of infecting tumor cells passaged through immune-compromised mice [22]. We tested the VCaP cell line directly from ATCC and confirmed that the distributed VCaP is positive for virus. To confirm that the infection of LAPC4 cells represents an infection that was present in early passage LAPC4 (as opposed to contamination from our laboratory), we obtained LAPC4 cells frozen down in 1998 as well as cells currently grown in the laboratory (i.e., from 2010) from the C. L. Sawyers lab at Memorial Sloan Kettering Cancer Center [17]. As shown in Figure S4, all LAPC4 cells tested from the Sawyers lab were positive for virus.

To further verify that the LAPC4 and VCaP cell lines are infected with integrated *Bxv*-1 provirus, we mapped multiple viral integration sites in each of these two lines using vectorette PCR [16]. Interestingly, viral integration sites were identified in the intronic regions of multiple genes in both LAPC4 and VCaP (Table 1). No common integration sites were identified between the two cell lines; however, we expect that the list of integration sites given in Table 1 is not exhaustive. Select integration sites were further verified from LAPC4 and VCaP using long-range PCR and primers that spanned the integration sites (Table 1).

**Table 1:** Integration sites for *Bxv-*1-like virus infecting LAPC4 and VCaP prostate cancer cell lines.

Line	Chromosome	Start*	Location
**LAPC4**	3**	3527870	Intron 2–3 of AC026188.1 Novel processed transcript
	9	25617692	
**VCaP**	1**†	245135029	Intron 1–2 of EF-hand calcium binding domain 2 (EFCAB2)
	1	234030449	
	4	71834780	Intron 2–3 of Mps one binder kinase activator-like 1A (MOBKL1A)
	7†	2550730	Intron 2–3 of AC092488.1 Known protein coding
	11	85540286	
	12	124181500	Intron 12–13 of Tectonic family member 2 (TCTN2)
	20	12058633	

*Based on Ensembl BLAT release 60- Nov 2010.

**Confirmed by PCR amplification and sequencing across integration sites.

†Located within 5 kb upstream or downstream of a known transcription start site.

### Infected cancer cell lines produce replication competent virus

**Figure 3:**
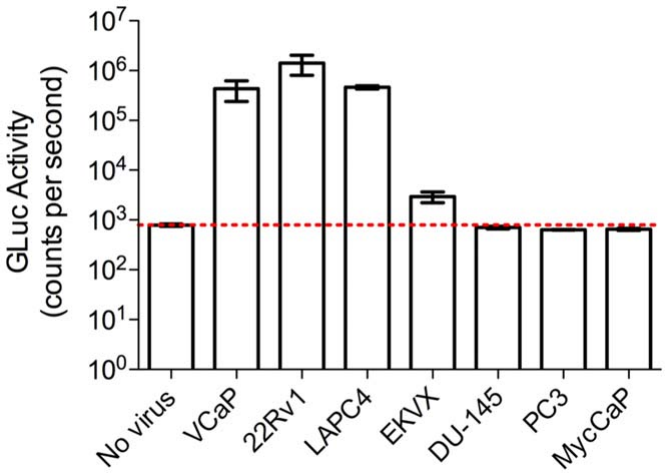
CWR22Rv1, LAPC4, VCaP and EKVX produce replication competent virus as indicated by *Gaussia* luciferase production in iGLuc-DERSE cells. 10 µl of media was assayed 2 days after exposure to 24 hr. cell culture supernatant from CWR22Rv1, LAPC4 and VCaP, and 13 days (and 3 passages) after exposure to supernatant from EKVX, DU145, MycCaP (murine prostate cancer cell line) and PC3. Red line indicates background luciferase expression as determined by a mock-infected control. Error bars represent standard error of the mean for 3 experiments.

To verify that the commonly used prostate cancer cell lines LAPC4 and VCaP contain integrated and replication-competent virus, we performed a viral infectivity assay. The indicator cell line, iGLuc-DERSE cells, will only secrete the *Gaussia* luciferase enzyme following infection with a replication-competent MLV. *Gaussia* luciferase activity was detected 48 hours after infection of iGLuc-DERSE cells with supernatant from the CWR22Rv1, LAPC4 and VCaP cell lines (Figure 3). Interestingly, a triplicate infection of iGLuc-DERSE cells with supernatant from the EKVX cell line did not show *Gaussia* luciferase activity until 13 days (and 3 passages) after infection (Figure 3). Further, only 2 of the 3 infections showed luciferase activity above background. Thus, the virus from EKVX cells apparently has very low infectivity, at least for the iGLuc-DERSE cell line. No *Gaussia* luciferase activity was detected from cells (passaged for a total of 13 days) exposed to supernatant from the DU145, MycCaP (a mouse prostate cancer cell line obtained from the C. L. Sawyers lab) or PC3 cell lines (Figure 3). CWR22Rv1 was previously reported to produce replication competent XMRV [4] but it has not been reported that LAPC4, VCaP and EKVX produce replication competent virus.

Out of concern that prostate cancer cell lines producing replication competent viruses could cross-contaminate other cell lines in our laboratory which are negative for MLVs, we tested current cultures in our laboratory for the presence of MLVs. We were surprised to find two instances where MLV-negative cell lines maintained in the laboratory (DU145, LNCaP) have become contaminated with MLVs (Figure S5). For example, when these cell lines were obtained directly from ATCC (LNCaP) or the NCI (DU145) they were negative for virus, indicating that the lines were contaminated at some point during culture in the laboratory. PCR analysis with XMRV-specific primers indicated that the contaminating virus was likely XMRV, potentially from culture alongside CWR22Rv1 (data not shown). These results suggest that if CWR22Rv1 cells are routinely cultured in a typical biomedical research laboratory setting (e.g. using standard Class II biosafety cabinets and procedures for cell culture in which two different cell lines are never present under the hood at the same time), that XMRV can infect and contaminate other cell lines.

## Discussion

Our study demonstrates that prostate cancer cell lines appear to have a propensity for infection by murine gammaretroviruses. We propose that this tendency may be due to the fact that most of the established prostate cancer cell lines were created by passage through immunocompromised mice. In fact, *Bxv*-1 is present in SCID and *nude* mice and tumor cells passaged through these animals can become infected with xenotropic MLVs [22]. Although the infection of human cell lines with murine gammaretroviruses has been previously described in the literature, what makes our current study particularly important is that prior to this study it was not known that multiple commonly used prostate cancer cell lines are contaminated with MLVs. In fact, we discovered during the course of our study that other cell lines maintained in the laboratory (DU145, LNCaP) have apparently become infected with XMRV in the laboratory.

In concordance with the data provided in the current study, in a recent study by Hue *et. al.*, which screened 411 human tumor cell lines from the Catalogue Of Somatic Mutations In Cancer (COSMIC) collection, EKVX was found to be positive for a xenotropic MLV-related virus by direct sequencing of viral *gag*, *pol* and *env* genes [8]. Furthermore, the Hue *et. al.* study tested all but 14 of the cell lines examined in the current study (with the exception of LAPC4, LNCaP, PrEC, VCaP, CWR22Rv1, RWPE, abl, PrSC, C4-2B, 957 E/h, PacMet UT1, MDA-PAC2b, DLD-1 and Hep3B) and likewise found them to be negative for MLV. Although the authors of the study did not attempt to obtain a full-length sequence of the virus contaminating EKVX or to demonstrate that the virus is replication competent, partial *pol* and *env* sequences from the Hue *et. al.* study (GenBank Accession #FR670589 and FR670597, respectively) match at 100% similarity to the near full-length viral sequence obtained in the present study (GenBank Accession #JF908817). We have now demonstrated that the virus contained in the EKVX cell line is replication competent (Figure 3), clusters in phylogenetic analysis with known xenotropic MLVs (Figure 2, Supporting Figure S3) and is 99% similar to a retrovirus previously isolated from the DG-75 B-lymphoblastoid cell line. Like all of the prostate cancer cell lines found to contain MLVs in the present study, the EKVX cell line was established by xenograft passage in *nude* mice [23].

There are several reported examples of retroviral infection modifying the biological properties of human cell lines. For example, the *in vivo* immunogenicity of cell lines has been shown to be strongly altered following retroviral infection after a single passage in *nude* mice [24]. Likewise, xenotropic MLVs acquired in cell lines used in HIV research after *in vivo* passage in immunocompromised mice were shown to alter the biological properties of HIV-1 [25]. Studies have also shown that MLV tends to integrate near transcription start sites (within 5 kb upstream or downstream) of actively transcribed genes [26]. Interestingly, two of the seven viral integration sites identified for VCaP are within 5 kb of a transcription start site (Table 1). An integration site located on Chromosome 1 is located approximately 2 kb downstream of the transcription start site of the EFCAB2 (EF-hand calcium binding domain 2) gene. Likewise, an integration site identified on Chromosome 7 is located approximately 1.4 kb upstream of LFNG (O-fucosylpeptide 3-beta-N-acetylglucosaminyltransferase), a gene involved in Notch signaling. It is not known what confounding effects the infection of commonly used prostate cancer cell lines with replication competent murine gammaretroviruses may have on experimental outcomes. Interestingly, it has been reported that XMRV proteins are more abundant in cell culture media of CWR22Rv1 cells than any human protein [4]. For reasons such as this, we suggest that cancer cell lines should undergo routine screening for contaminating MLVs, much like the current practice of routine testing of cultured cells for mycoplasma.

## Supporting Information

Text S1 Supporting methods.
(DOC)

Figure S1 Determining the sensitivity of the Pan-MLV and XMRV-specific PCR assays. Serial dilutions of CWR22Rv1 genomic DNA were spiked into 100 ng of LNCaP (MLV-negative) genomic DNA and used as a template for PCR. (**A**) Pan-MLV primers (**B**) XMRV-specific primers. Samples were tested in duplicate: 1 ng CWR22Rv1 (lane 1–2), 0.1 ng CWR22Rv1 (lane 3–4), 0.01 ng CWR22Rv1 (lane 5–6), 0.001 ng CWR22Rv1 (lane 7–8), negative control (lane 9–10). L =  molecular weight ladder.
(TIF)

Figure S2 Testing MLV-positive cell lines for the presence of contaminating mouse DNA by PCR assay for IAP. Lane 1 =  C57BL/6J mouse genomic DNA (positive control), lane 2 =  CWR22Rv1, lane 3 =  LAPC4, lane 4 =  VCaP, lane 5 =  EKVX, lane 6 =  negative control. All cell lines tested were negative for contaminating mouse DNA. L =  molecular weight ladder.
(TIF)

Figure S3 Phylogenetic analysis of virus genomes from LAPC4, VCaP and EKVX cell lines. The phylogenetic tree represents known endogenous MLV sequences, including polytropic (Pmv), modified polytropic (Mpmv) and xenotropic (Xm) viruses. This tree represents only endogenous MLVs for which the full proviral sequence is known, which is likely only a small portion of all endogenous MLVs present in mice. Exogenous MLVs (CAS-BR-E, AKV, MLMCG and Friend) are also included for comparison.
(TIF)

Figure S4 Testing early passage LAPC4 for presence of virus. The pan-MLV primers described in Figure 1 were used to test LAPC4 cells from our laboratory (lane 1), early passage LAPC4 frozen down in 1998 from the C. Sawyers lab (lane 2), LAPC4 cells frozen down in 2010 from the C. Sawyers lab (lane 3), and a mock DNA extraction negative control (lane 4). All LAPC4 cells analyzed were positive for virus.
(TIF)

Figure S5 Examples of MLV-negative prostate cancer cell lines that have become contaminated with MLV during serial passage by cell culture in the laboratory. Prostate cancer cell lines obtained directly from the NCI (DU145) or ATCC (LNCaP) are negative when stained with MLV30 and MLV70 antisera. These lines were unexpectedly found to be positive for virus after serial passage in the lab.
(TIF)

Table S1 Complete results of IHC and PCR on 72 human cell lines.
(DOC)

Table S2 Primers used to sequence viral genomes.
(DOC)
