# Supplementary material for: Identification of Replication Competent Murine Gammaretroviruses in Commonly Used Prostate Cancer Cell Lines
Source: PLoS One. 2011 Jun 17;6(6):e20874. doi: 10.1371/journal.pone.0020874 (PMC3117837; doi:10.1371/journal.pone.0020874)
Supplement: Text S1 — Supporting methods. (DOC) [file pone.0020874.s001.doc]

**Supporting Methods**

**Cell lines**. The cell lines used in the construction of the cell line TMA and for DNA extraction were obtained from the following sources: the NCI-60 (excluding SF268 and MDA-MB-468, which were either unavailable or we were unable to culture) was obtained from the NCI-Frederick (http://dtp.nci.nih.gov/docs/misc/common_files/cell_list.html); LNCaP, VCaP, CWR22Rv1, Hep3B and DLD-1 were obtained from the American Type Culture Collection (ATCC); PrEC and PrSC cells were obtained from Lonza (Basel, Switzerland); LAPC4, RWPE, C4-2B, 957E*/*hTERT, PacMetUT1, and MDA-PAC2b were obtained from J.T. Isaacs (Johns Hopkins University); LNCaP-abl cells were obtained from Z. Culig (University of Innsbruck). Early passage LAPC4 cells were obtained from C.L. Sawyers (Memorial Sloan-Kettering Cancer Center).

**Cell fixation and processing for TMA construction**. Cell lines were grown to 80-100% confluence, harvested by trypsinization, and washed once with PBS. Pellets were then resuspended in a small volume of PBS and pipetted into a 5 mL cryovial that had a small plug of solidified 2% agarose at the bottom. The cryovial was then centrifuged in a swinging bucket rotor to pellet the cells. The PBS was aspirated off and replaced with 10% formalin. The cells are thus fixed as a “solid” plug without the need for a supporting (agarose) matrix. Pellets were approximately 5-10 mm thick. After 24 hours of fixation, the bottom of the cryovial was carefully cut off such that the plugs could be deposited in a tissue cassette for standard paraffin embedding and processing. The resulting cell line blocks were then used for TMA construction. Additional details of the process of cell line embedding and TMA generation will be presented in a separate publication (manuscript in process).

**Long-range PCR and sequencing of viral genomes**. Full-length (LAPC4, VCaP) or near full-length (EKVX) viral genomes were amplified from genomic DNA preparations of the LAPC4, VCaP and EKVX cell lines in two overlapping ~4-4.5 kb fragments via long-range PCR using the LongAmp® Taq PCR Kit (New England Biolabs) for LAPC4 and VCaP or the LongRange PCR Kit (Qiagen) for EKVX and the manufacturer’s recommended protocol. The primer sets used for viral genome amplification as well as for genome sequencing are listed in Table S2. The PCR cycling conditions were as follows for LAPC4 and VCaP: Initial denature at 94°C for 30 sec., 35 cycles of 94°C for 30 sec., 54°C for 30 sec. and 65°C for 4 min., 65°C for 10 min., 4°C hold. The PCR cycling conditions were as follows for EKVX: Initial denature at 93°C for 3 min., 38 cycles of 93°C for 15 sec., 53°C for 30 sec. and 68°C for 4 min., 68°C for 10 min., 4°C hold. PCR products were extracted from a 1.2% agarose gel using the QIAquick Gel Extraction Kit (Qiagen) and sequenced directly as overlapping sequences and full-length consensus sequences were assembled using the Sequencher 4.1.4 program (Gene Codes).
