# Supplementary figures and images for: Identification of Replication Competent Murine Gammaretroviruses in Commonly Used Prostate Cancer Cell Lines
Source: PLoS One. 2011 Jun 17;6(6):e20874. doi: 10.1371/journal.pone.0020874 (PMC3117837; doi:10.1371/journal.pone.0020874)

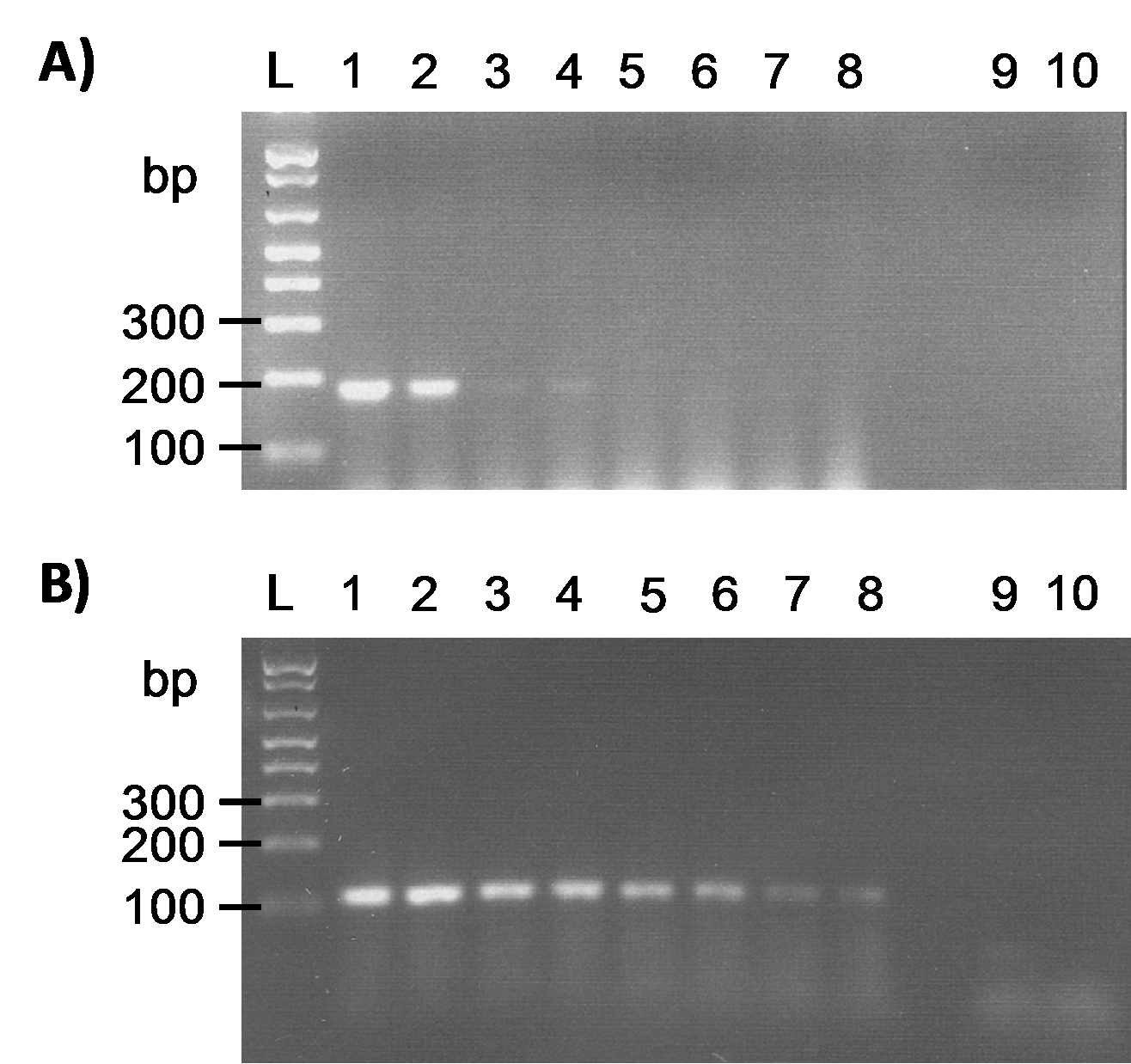

Supplement: Figure S1 — Determining the sensitivity of the Pan-MLV and XMRV-specific PCR assays. Serial dilutions of CWR22Rv1 genomic DNA were spiked into 100 ng of LNCaP (MLV-negative) genomic DNA and used as a template for PCR. (A) Pan-MLV primers (B) XMRV-specific primers. Samples were tested in duplicate: 1 ng CWR22Rv1 (lane 1–2), 0.1 ng CWR22Rv1 (lane 3–4), 0.01 ng CWR22Rv1 (lane 5–6), 0.001 ng CWR22Rv1 (lane 7–8), negative control (lane 9–10). L = molecular weight ladder. (TIF) [file pone.0020874.s002.tif]

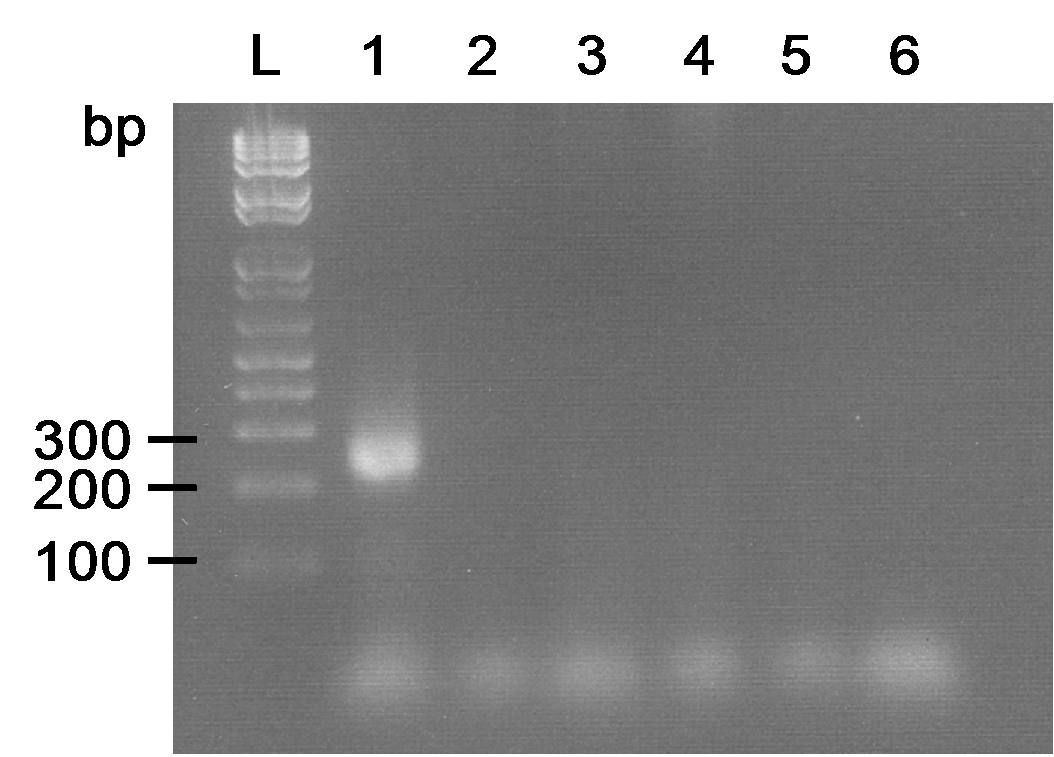

Supplement: Figure S2 — Testing MLV-positive cell lines for the presence of contaminating mouse DNA by PCR assay for IAP. Lane 1 = C57BL/6J mouse genomic DNA (positive control), lane 2 = CWR22Rv1, lane 3 = LAPC4, lane 4 = VCaP, lane 5 = EKVX, lane 6 = negative control. All cell lines tested were negative for contaminating mouse DNA. L = molecular weight ladder. (TIF) [file pone.0020874.s003.tif]

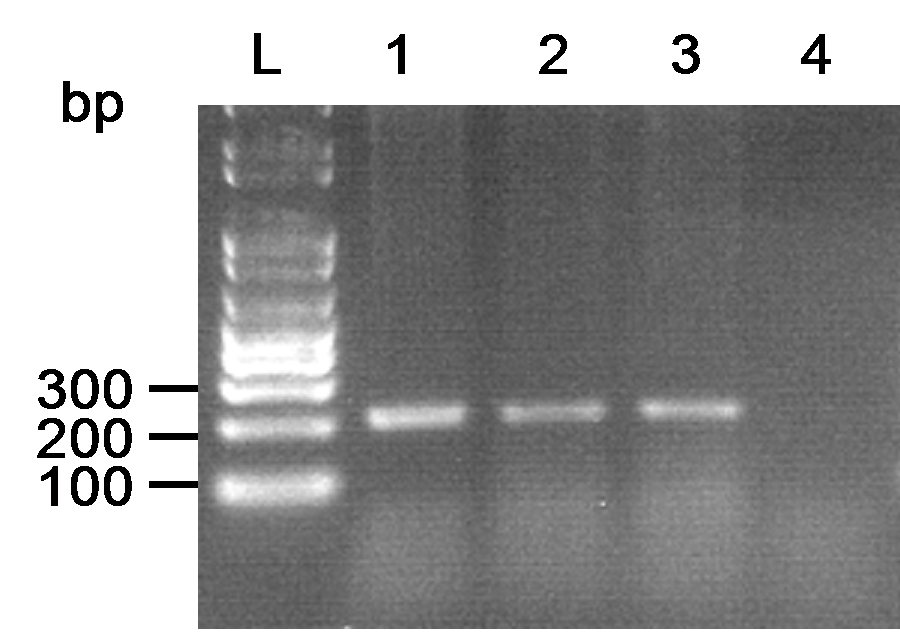

Supplement: Figure S4 — Testing early passage LAPC4 for presence of virus. The pan-MLV primers described in Figure 1 were used to test LAPC4 cells from our laboratory (lane 1), early passage LAPC4 frozen down in 1998 from the C. Sawyers lab (lane 2), LAPC4 cells frozen down in 2010 from the C. Sawyers lab (lane 3), and a mock DNA extraction negative control (lane 4). All LAPC4 cells analyzed were positive for virus. (TIF) [file pone.0020874.s005.tif]

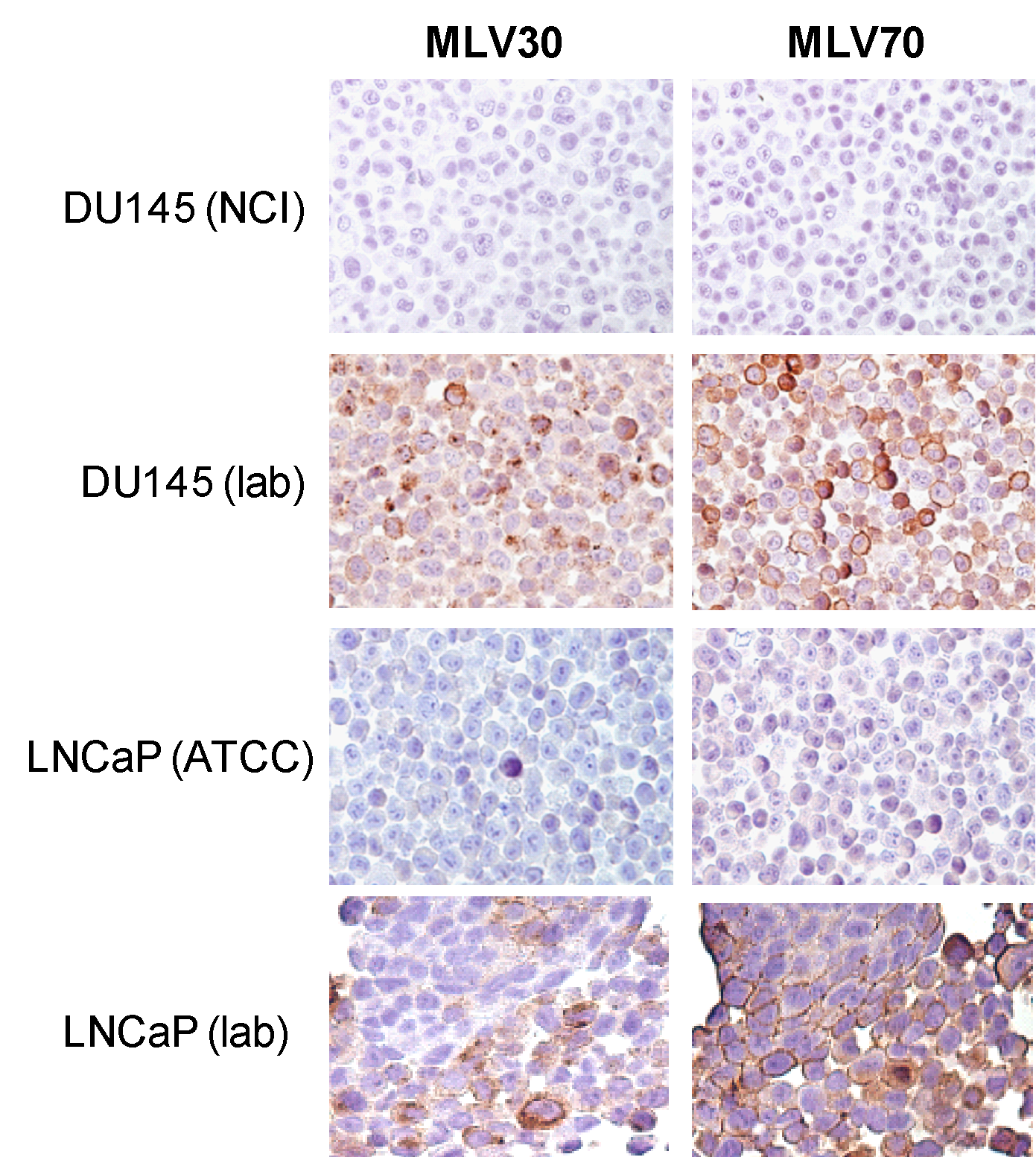

Supplement: Figure S5 — Examples of MLV-negative prostate cancer cell lines that have become contaminated with MLV during serial passage by cell culture in the laboratory. Prostate cancer cell lines obtained directly from the NCI (DU145) or ATCC (LNCaP) are negative when stained with MLV30 and MLV70 antisera. These lines were unexpectedly found to be positive for virus after serial passage in the lab. (TIF) [file pone.0020874.s006.tif]
