# Supplementary material for: Identification of Replication Competent Murine Gammaretroviruses in Commonly Used Prostate Cancer Cell Lines
Source: PLoS One. 2011 Jun 17;6(6):e20874. doi: 10.1371/journal.pone.0020874 (PMC3117837; doi:10.1371/journal.pone.0020874)
Supplement: Table S1 — Complete results of IHC and PCR on 72 human cell lines. (DOC) [file pone.0020874.s007.doc]

**Table S1.** Complete results of IHC and PCR on 72 human cell lines.

| **Cell Line Name** | **Origin** | **IHC MLV30** | **IHC MLV70** | **PCR (MLV primers)** | **PCR (XMRV primers)** |
| --- | --- | --- | --- | --- | --- |
| CCRF-CEM | Leukemia | **⁻** | **⁻** | **⁻** | **⁻** |
| HL-60(TB) | Leukemia | **⁻** | **⁻** | **⁻** | **⁻** |
| K-562 | Leukemia | **⁻** | **⁻** | **⁻** | **⁻** |
| MOLT-4 | Leukemia | **⁻** | **⁻** | **⁻** | **⁻** |
| RPMI-8226 | Leukemia | **⁻** | **⁻** | **⁻** | **⁻** |
| SR | Leukemia | **⁻** | **⁻** | **⁻** | **⁻** |
| A549 | Non-Small Cell Lung | **⁻** | **⁻** | **⁻** | **⁻** |
| EKVX | Non-Small Cell Lung | ⁺ | ⁺ | ⁺ | **⁻** |
| HOP-62 | Non-Small Cell Lung | **⁻** | **⁻** | **⁻** | **⁻** |
| HOP-92 | Non-Small Cell Lung | **⁻** | **⁻** | **⁻** | **⁻** |
| NCI-H226 | Non-Small Cell Lung | **⁻** | **⁻** | **⁻** | **⁻** |
| NCI-H23 | Non-Small Cell Lung | **⁻** | **⁻** | **⁻** | **⁻** |
| NCI-H322M | Non-Small Cell Lung | **⁻** | **⁻** | **⁻** | **⁻** |
| NCI-H460 | Non-Small Cell Lung | **⁻** | **⁻** | **⁻** | **⁻** |
| NCI-H522 | Non-Small Cell Lung | **⁻** | **⁻** | **⁻** | **⁻** |
| COLO 205 | Colon | **⁻** | **⁻** | **⁻** | **⁻** |
| DLD-1 | Colon | **⁻** | **⁻** | **⁻** | **⁻** |
| HCC-2998 | Colon | **⁻** | **⁻** | **⁻** | **⁻** |
| HCT-116 | Colon | **⁻** | **⁻** | **⁻** | **⁻** |
| HCT-15 | Colon | **⁻** | **⁻** | **⁻** | **⁻** |
| HT29 | Colon | **⁻** | **⁻** | **⁻** | **⁻** |
| KM12 | Colon | **⁻** | **⁻** | **⁻** | **⁻** |
| SW-620 | Colon | **⁻** | **⁻** | **⁻** | **⁻** |
| SF-295 | CNS | **⁻** | **⁻** | **⁻** | **⁻** |
| SF-539 | CNS | **⁻** | **⁻** | **⁻** | **⁻** |
| SNB-19 | CNS | **⁻** | **⁻** | **⁻** | **⁻** |
| SNB-75 | CNS | **⁻** | **⁻** | **⁻** | **⁻** |
| U251 | CNS | **⁻** | **⁻** | **⁻** | **⁻** |
| LOX IMVI | Melanoma | **⁻** | **⁻** | **⁻** | **⁻** |
| MALME-3M | Melanoma | **⁻** | **⁻** | **⁻** | **⁻** |
| M14 | Melanoma | **⁻** | **⁻** | **⁻** | **⁻** |
| MDA-MB-435 | Melanoma | **⁻** | **⁻** | **⁻** | **⁻** |
| SK-MEL-2 | Melanoma | **⁻** | **⁻** | **⁻** | **⁻** |
| SK-MEL-28 | Melanoma | **⁻** | **⁻** | **⁻** | **⁻** |
| SK-MEL-5 | Melanoma | **⁻** | **⁻** | **⁻** | **⁻** |
| UACC-257 | Melanoma | **⁻** | **⁻** | **⁻** | **⁻** |
| UACC-62 | Melanoma | **⁻** | **⁻** | **⁻** | **⁻** |
| IGR-OV1 | Ovarian | **⁻** | **⁻** | **⁻** | **⁻** |
| OVCAR-3 | Ovarian | **⁻** | **⁻** | **⁻** | **⁻** |
| OVCAR-4 | Ovarian | **⁻** | **⁻** | **⁻** | **⁻** |
| OVCAR-5 | Ovarian | **⁻** | **⁻** | **⁻** | **⁻** |
| OVCAR-8 | Ovarian | **⁻** | **⁻** | **⁻** | **⁻** |
| NCI/ADR-RES | Ovarian | **⁻** | **⁻** | **⁻** | **⁻** |
| SK-OV-3 | Ovarian | **⁻** | **⁻** | **⁻** | **⁻** |
| 786-0 | Renal | **⁻** | **⁻** | **⁻** | **⁻** |
| A498 | Renal | **⁻** | **⁻** | **⁻** | **⁻** |
| ACHN | Renal | **⁻** | **⁻** | **⁻** | **⁻** |
| CAKI-1 | Renal | **⁻** | **⁻** | **⁻** | **⁻** |
| RXF 393 | Renal | **⁻** | **⁻** | **⁻** | **⁻** |
| SN12C | Renal | **⁻** | **⁻** | **⁻** | **⁻** |
| TK-10 | Renal | **⁻** | **⁻** | **⁻** | **⁻** |
| UO-31 | Renal | **⁻** | **⁻** | **⁻** | **⁻** |
| 957 E/h | Prostate | **⁻** | **⁻** | **⁻** | **⁻** |
| abl | Prostate | **⁻** | **⁻** | **⁻** | **⁻** |
| C4-2B | Prostate | **⁻** | **⁻** | **⁻** | **⁻** |
| CWR22Rv1 | Prostate | ⁺ | ⁺ | ⁺ | ⁺ |
| DU-145 | Prostate | **⁻** | **⁻** | **⁻** | **⁻** |
| LAPC4 | Prostate | ⁺ | ⁺ | ⁺ | **⁻** |
| LNCaP | Prostate | **⁻** | **⁻** | **⁻** | **⁻** |
| MDA-PAC2b | Prostate | **⁻** | **⁻** | **⁻** | **⁻** |
| PacMet UT1 | Prostate | **⁻** | **⁻** | **⁻** | **⁻** |
| PC-3 | Prostate | **⁻** | **⁻** | **⁻** | **⁻** |
| PrEC | Prostate | **⁻** | **⁻** | **⁻** | **⁻** |
| PrSC | Prostate | **⁻** | **⁻** | **⁻** | **⁻** |
| RWPE | Prostate | **⁻** | **⁻** | **⁻** | **⁻** |
| VCaP | Prostate | ⁺ | ⁺ | ⁺ | **⁻** |
| MCF7 | Breast | **⁻** | **⁻** | **⁻** | **⁻** |
| MDA-MB-231 | Breast | **⁻** | **⁻** | **⁻** | **⁻** |
| HS 578T | Breast | **⁻** | **⁻** | **⁻** | **⁻** |
| BT-549 | Breast | **⁻** | **⁻** | **⁻** | **⁻** |
| T-47D | Breast | **⁻** | **⁻** | **⁻** | **⁻** |
| Hep3B | Liver | **⁻** | **⁻** | **⁻** | **⁻** |
