# Supplementary material for: Identification of Replication Competent Murine Gammaretroviruses in Commonly Used Prostate Cancer Cell Lines
Source: PLoS One. 2011 Jun 17;6(6):e20874. doi: 10.1371/journal.pone.0020874 (PMC3117837; doi:10.1371/journal.pone.0020874)
Supplement: Table S2 — Primers used to sequence viral genomes. (DOC) [file pone.0020874.s008.doc]

**Table S2.** Primers used to sequence viral genomes.

| **Cell Line** | **Amplification Primers*** | **Location**** | **Sequence 5'-3'** | **Sequencing Primers***** | **Sequence 5'-3'** |
| --- | --- | --- | --- | --- | --- |
| LAPC4,  VCaP | PanVirus-F1 | 5’ LTR/470 | TCCTTGGGAGGGTCTCCTC | Virus-1012F | ATCTGCCCTTTACCCTGCTCTTA |
|  | PanVirus-R1 | pol/4700 | AGCYTCTTACCTTCTGCCAT | Virus-1861F | GTTAGAGCGGTTAGAAGACT |
|  | PanVirus-F3 | pol/4530 | CAGACGCCGACCACACCTGGTA | Virus-3403R | GGCAGATTTGGGCTTTCTTG |
|  | PanVirus-R2 | 3’ LTR/8518 | GGGCGACTCAGTCWATCGGA | Virus-5034F | GTACAAGTACCTCCTGGTGT |
|  |  |  |  | Virus-5947F | CAAACAGCTAACGCTACCTC |
|  |  |  |  | Virus-7495R | CACCCTTGTCCTGATTCGAAC |
|  |  |  |  |  |  |
| EKVX | PanVirus-F2 | 5’ LTR/37 | CCGTGTTCCCAATAAAGCCT | Virus-736F | ACGTCAAGAAGAGACGCTGGGT |
|  | PanVirus-R1 | pol/4311 | see above | Virus-2182R | CAGTGTCCCTTTTCTTTGCAG |
|  | PanVirus-F3 | pol/4141 | see above | Virus-2897R | TAGTCCCTGGTTTCTTAACG |
|  | PanVirus-R2 | 3’LTR/8145 | see above | Virus-2723F | GGACTGGCAGTTCGCCAAG |
|  |  |  |  | Virus-3828R | GCAATGGCTGCTACCATCCG |
|  |  |  |  | Virus-5034F | see above |
|  |  |  |  | Virus-7495R | see above |

* Viral genomes were amplified in two overlapping ~4.5 kb fragments. ** Location of primer (gene/nucleotide position) relative to *Bxv*-1 (see Jern et. al. 2007 PLOS Genetics 3: e183) for LAPC4 and VCaP or DG-75 (GenBank Accession #AF221065) for EKVX. *** In addition to the primers listed for genome amplification, the primers listed were used to sequence viral genomes.
